# Zirconia crowns cemented on titanium bars using CAD/CAM: a five-year follow-up prospective clinical study of 9 patients

**DOI:** 10.1186/s12903-019-0988-x

**Published:** 2019-12-19

**Authors:** Antonio Scarano, Marco Stoppaccioli, Tommaso Casolino

**Affiliations:** 1 Department of Medical, Oral and Biotechnological Sciences, University “G. D’Annunzio” of Chieti-Pescara, Via Dei Vestini, 31, 66100 Chieti, Italy; 2 Zirconia Implant Research Group (Z.I.R.G), International Academy of Ceramic Implantology, Silver Spring, USA; 3 L’Aquila, Italy; 4 Termoli, Italy

**Keywords:** CAD/CAM, Zirconia, Dental implants, Digital workflow, Toronto bridge, Computerized workflow

## Abstract

**Background:**

The purpose of this prospective clinical study was to evaluate clinical results of the passive fit of the substructure in the Toronto bridge and the chipping or delamination of the ceramic veneering on the zirconia-support, after 5 years, in nine patients rehabilitated with zirconia crowns cemented on titanium bars using CAD/CAM technology.

**Methods:**

A total of nine healthy patient fully edentulous in the upper and lower jaws with non-contributory past medical anamnesis needing full fixed total prosthesis maxilla and mandible were included in this clinical study, where a total 9 mandibles and 9 jaws were treated. The inclusion criteria in order for a patient to participate in the study were: a signed consent form, fully edentulous in the upper and lower jaws, required a full fixed total prosthesis restoration. The exclusion criteria were age limitation of less than 18 years old, chemotherapy, head and neck radiation therapy, diabetes or periodontal disease, smoking and severe illness. All patients received zirconia crowns cemented on titanium bars using CAD/CAM technology. The primary outcome of this study was to examine the survival rate of the zirconia crowns cemented on titanium bars using CAD/CAM technology during the observation period. Any chipping or delamination of the zirconia crowns of the restorations was considered as failure. The secondary outcome was to evaluate the passive fit of the substructure on the implants, loose of occlusal screws, implant survival and satisfactory occlusion.

**Results:**

In 5 years of follow-up no evidence of chipping or delamination of the ceramic veneering on the zirconia crown supported were observed. Fifteen finished protesis (93.75%) showed satisfactory occlusion and only one case (6.25%) required significant occlusal adjustment. During the first year recall all bars were stable (100%) no mobility of protheses was recorded. After 5 years all bars were stable (100%) and no mobility of protheses was recorded.

**Conclusion:**

The computerized workflow for the process of building bar and prosthesis ensures reproducible results and excellent adaptation and passive insertion of them, as well as conditions for avoiding mechanical complications and guarantees stability of screw-implant abutments.

## Background

The long-term predictability of the restoration involves several aspects concerning the implant surface [1], surgical techniques [2, 3] and the anchorages used. Screw-retained prosthesis, cemented, or a combined approach have been proposed as prosthetic connections between the implant and the restoration. The screwed prothesis has the advantage of the possibility of later removal for eventual restoration, repair, treatment of the implants, retightening of the abutment screw and hygiene measurements [4]. Frequently, the position of the screw hole of tilted implants may produce complications in terms of occlusion and aesthetic outcome [5]. Moreover, it might lead to fracture of the porcelain if the metal framework design does not support the porcelain around the access hole [6].

A cemented approach for implant restorations has been proposed to improve the aesthetic outcome in tilted implants by means of customized abutments [7]. This technique presents many benefits over screw-retained restorations. These advantages include higher potential for achieving a passive fit of the superstructure, easy fabrication, improved aesthetic results and an easier positioning in posterior regions of the oral cavity [8, 9].

However, this technique has some disadvantages such as difficulty in removing the cement excess around the implant restoration. High priority should be given to the removal of excess cement after cementation in the implant-mucosal interface, to avoid bleeding on probing in most cases and suppuration in some [10].

The fixed prosthesis on Toronto Bridge implants or abutment-hybrid overdenture, have been proposed in order to overcome problems of both types of screw or cemented restorations while benefiting from their merits [11]. The Toronto Bridge procedure mixes the advantages of screw- and cement-retained prostheses by a screwed in mesostructure and milled abutments for the cementation of single or multiple superstructures. Individual crowns are cemented on the superstructures with a pink characterization or gingiva-coloured laboratory composite or porcelain to simulate the soft tissues. The fixed prosthesis on Toronto Bridge implants takes its name from the Canadian city where it was presented for the first time by Zarb GA [12] in early 1980s.

Full tooth therapy with Toronto Bridge in the totally edentulous patients is frequently indicated for implant therapy. The fixed prosthesis on Toronto Bridge implants is a fixed full total prosthesis with flange that can replace an entire dental arch and is a good alternative solution to be taken into consideration. It usually consists of 10/12 teeth per arch and is directly secured, by abutments, to dental osseointegrated titanium implants. In many cases, immediate load implants can be used, and for this purpose, the All-on-4 surgical protocol is one of the most used [13–15]. The Toronto Bridge is used when the patient requires a supported implant with need of a good support of the lips with a flange. Today the term Toronto Bridge has taken on a broader meaning, including all cases of full-arch rehabilitation with reconstruction of hard and soft tissues, including cases with tilted implants. Toronto bridge technique or abutment-hybrid overdenture have a bar on which individual crowns can be cemented and pink composite or gingiva-colored porcelain is used for mimicking the soft tissues.

Computer-aided design and computer-aided manufacturing (CAD/CAM) systems for the design and manufacture of Toronto bridges are now used in clinical practice. The CAD/CAM compound prostheses were formerly introduced in clinical practice for single elements over 30 years ago, and advancements in technology now permit the manufacture of full or multi-unit rehabilitations [16]. Nowadays, digital impression with intraoral scanning or indirect digitization of casts derived from conventional impressions, can generate a stereolithography (STL) [17]. The intraoral situation can be moved to a virtual ‘cast-free working environment and, in case of necessity, physical casts can be made from the STL files [18]. The intraoral scanner (IOS) is used to transform conventional prosthetic workflows with the possibility of immediately obtaining digital models of the arches. The IOS are also used for the design and fabrication of implant-supported monolithic translucent zirconia crowns cemented on customized hybrid abutments [19]. The classic Toronto have a titanium bar with teeth and resin flange, this prosthesis has a good prognosis, but it is, unfortunately, not equally valid over time from an aesthetic point of view, and acrylic resin does not allow an adequate resistance response when the prosthesis is subjected to a load [20]. To avoid this, in the present study, we have used a cemented zirconia superstructure above a milled metal substructure.

The aim of this prospective clinical study was to evaluate clinical results after 5 years in nine patients rehabilitated with Toronto prostheses and zirconia crowns cemented on titanium bars using CAD/CAM compound prostheses.

## Methods

**Fig. 1:**
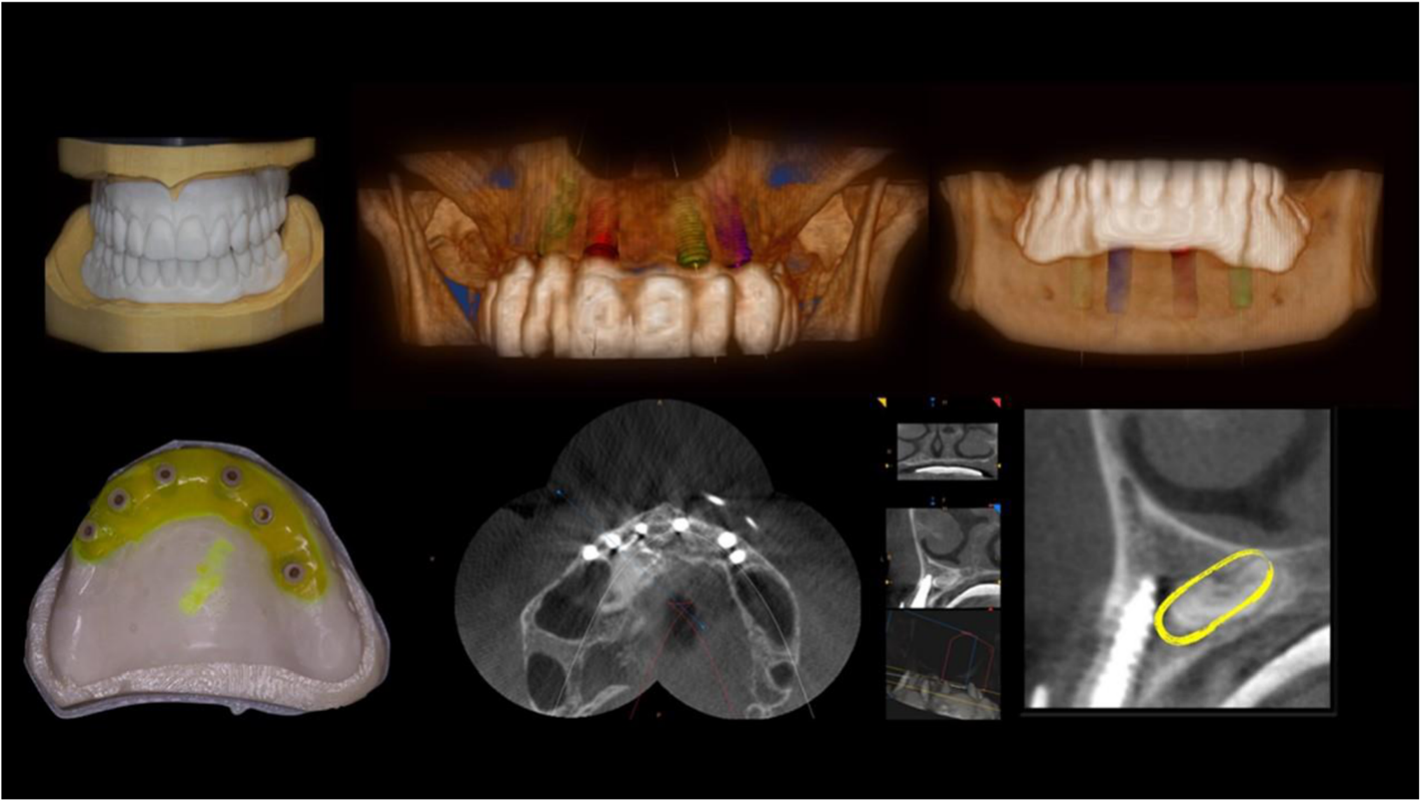
Initial case. Radiographic moulds, Implant planning through software (Implant3D), surgical moulds and post-surgical X-Ray. Yellow circle highlights the Impacted canine

**Fig. 2:**
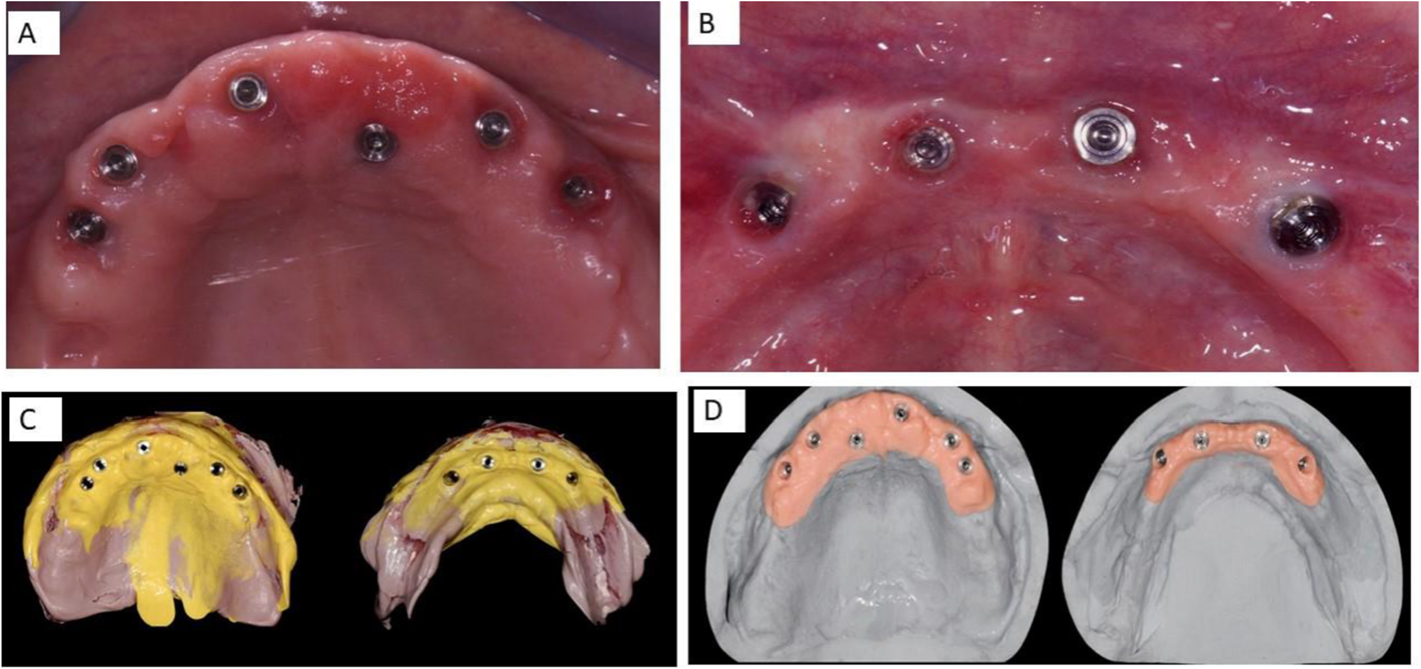
**a** The Upper arch. Occlusal view showing the six Intra-Lock System® implants with FlatOne® abutment, one implant was placed palatally to avoid extraction of impacted canine (The patient refused this surgery). **b** Lower arch with four Intra-Lock System® implants with FlatOne® abutment. **c** Impressions. **d** Master cast with silicone gingiva

**Fig. 3:**
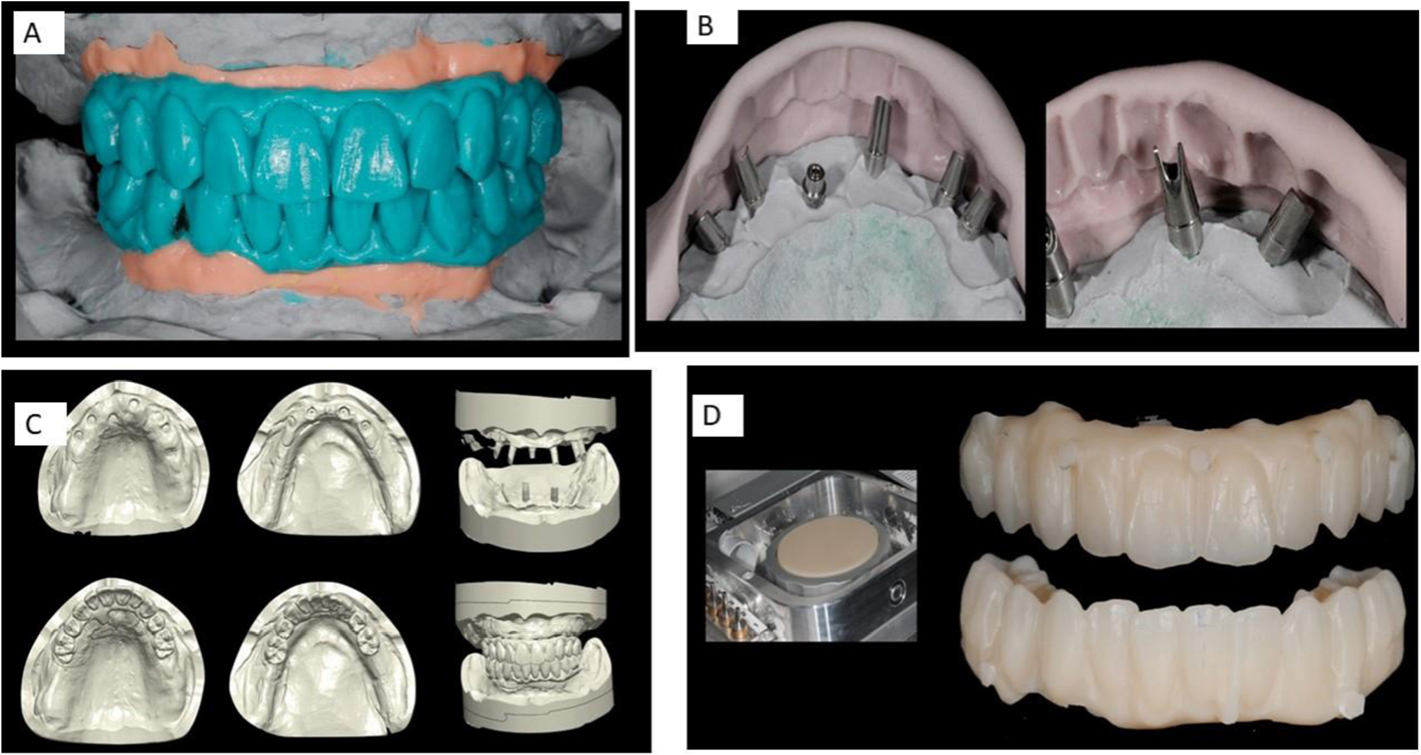
**a** Diagnostic Project designed with an analog wax-up. **b** Preparation of the prosthetic components for the temporary prosthesis. **c** Wax-up acquisition through a 3D scanner and planning of the temporary prosthesis using the modelling software. **d** Creation of a temporary upper and lower prosthesis through a numerically controlled milling machine with use of long-duration resins

The present prospective clinical study was based in a private practice in Termoli (Italy), in full accordance with ethical principles, including the World Medical Association Declaration of Helsinki and the additional requirements of Italian law. Furthermore, the University of Chieti-Pescara, Italy, classified the study to be exempt from ethical review as it carries only negligible risk and involves the use of existing data that contains only non-identifiable data about human beings. All patients signed a written informed consent form. A total of nine healthy patients fully edentulous in the upper and lower jaws with non-contributory past medical anamnesis needing full fixed total prosthesis (5 women and 4 men, all non-smokers, mean age 51 years, range 47–69 years) were included in this study, where a total 9 mandibles and 9 jaws were treated. A total of 18 superstructures were madefrom a prefabricated CAD/CAM framework, connected directly to the implants, using flat abtments. The patients were selected for full fixed total prosthesis and signed a written informed consent. The inclusion criteria in order for a patient to participate in the study were: a signed consent form, fully edentulous in the upper and lower jaws, required a full fixed total prosthesis restoration. The exclusion criteria were age limitation of less than 18 years old, chemotherapy, head and neck radiation therapy, diabetes or periodontal disease, smoking and severe illness. The primary outcome of this study was to examine the survival rate of the zirconia crowns cemented on titanium bars using CAD/CAM technology during the observation period. Any chipping or delamination of the ceramic veneering on zirconia-supports zirconia crowns of the restorations was considered as failure. The secondary outcome was to evaluate the passive fit of the substructure on the implants, loose of occlusal screws, implant survival and satisfactory occlusion. At the initial visit, all candidates underwent a clinical and occlusal evaluation, and orthopanoramic radiographs were evaluated. A three-dimensional cone beam computed tomography scan (CBCT) (Vatech Ipax 3D PCH-6500, Fort Lee, NJ USA) was performed to investigate clinically evident pathologies such as residual teeth, cists, height and thickness of the bone ridge. Before implant surgery, rinses of chlorhexidine digluconate solution 0.2% for 2 min (Curaden Healthcare S.p.A., Saronno, Italy) were administered to the patients. Local anaesthesia was performed using Articaine® (Pierrel, Milano Italy) with epinephrine 1:100.000. A total 90 implants were used, each mandible received four implants while the jaws received six dental implants (Intra-Lock International, Inc. - Florida, USA) inserted according to manufacturer’s recommendations. The procedure consisted of a computer guided surgery (Figs. 1 and 2). The case was planned to use the Implant3d (Milan, Italy) surgical planning software package. The radiographic guide, that duplicates the future prosthesis, allows the prediction of the diameter and length, positioning and orientation of the dental implants to anticipate the dimensions, locations and axes of the implants and abutments. The treatment planning software allows maximal exploitation of the available bone volume. One investigator (A.S) performed all virtual planning. Surgical guides were designed and exported from the virtual planning and then 3D printed, using a printer (Isomed, Padova, Italy). The Implants were placed with flapless technique. All guides were produced using transparent resin with thickness of 3 mm. Each operator visually checked guides for fit. Minor adjustments were permitted to the outer surface, in order to ensure full access of the surgical handpiece. After a healing period of 4 months, a healing screw was placed. During this phase a percussion test on the implants was carried out, and a control radiograph was taken for evaluation of grade of osseointegration of the implants. After another 2 weeks a Flatone® flat abutment (Intra-Lock- Salerno-Italy) was placed and definitively torqued to 30Ncm according to the manufacturer’s recommendation using a torque control system and an impression was taken (Fig. 3b).

**Fig. 4:**
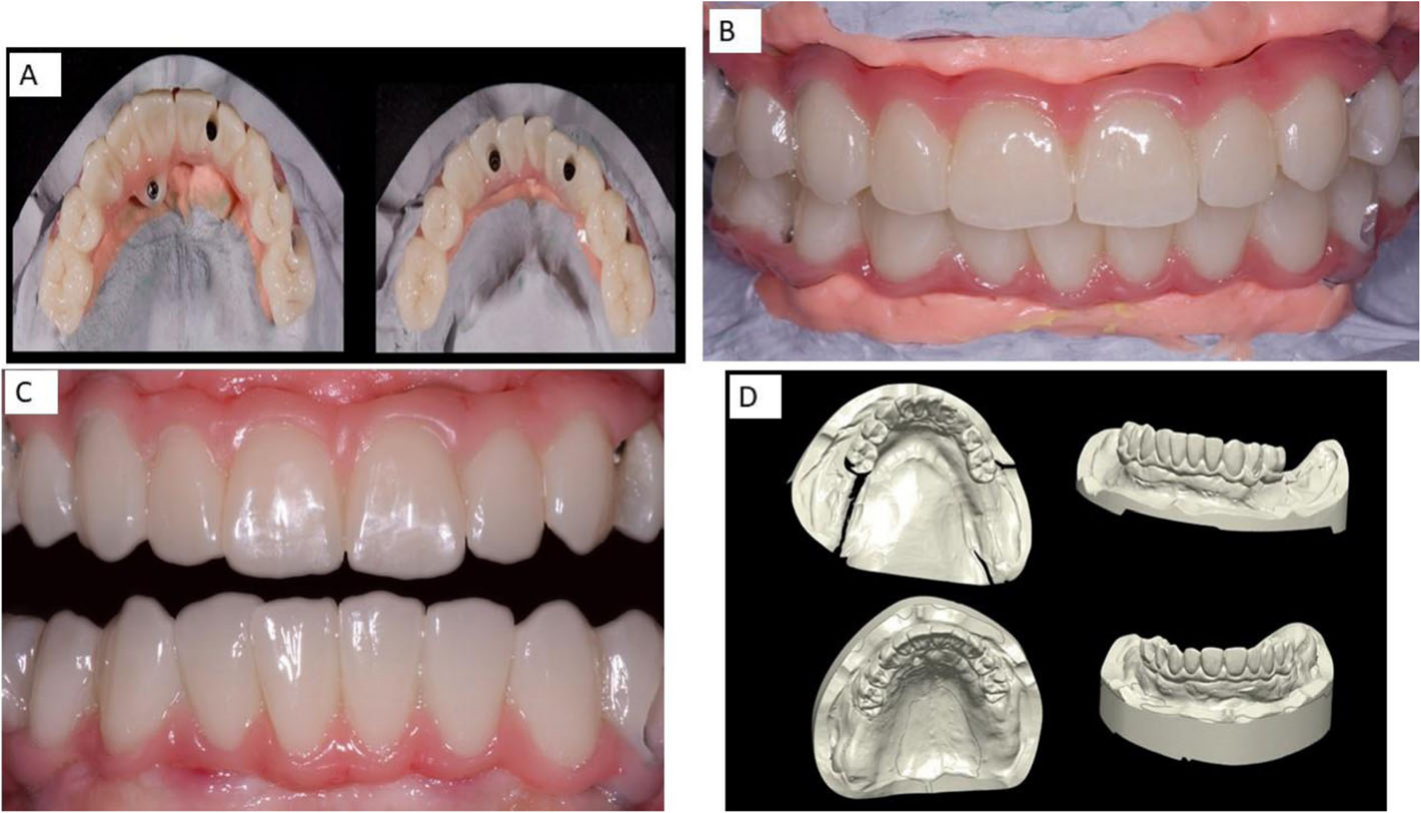
**a** Temporary prosthesis completed with a pink composite material. **b** Temporary prosthesis. **c** Temporary prosthesis in the mouth. **d** Temporary functionalized scanned prosthesis

**Fig. 5:**
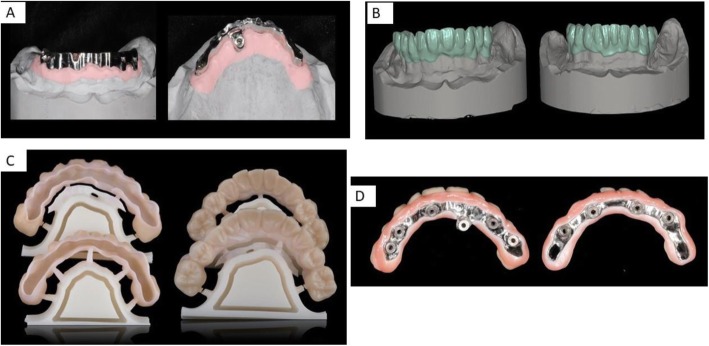
**a** Milled bars designed with systematic CAD/CAM. **b** Zirconia frame design. **c** Just zirconia frame. **d** View of the final prosthesis

Flatone® flat abutment systems, adopted to design the bars, improve the level of precision (Figs. 2 and 3). Digitization of casts derived from conventional impression made from polyeter material. Also, the models were developed by the laboratory and mounted on an articulator to establish the interarch relationship (Figs. 3 and 4) and then reacquired on the digital platform (Figs. 3 and 5).

After entering the data, with a dedicated software, titanium bars were designed by the same company supplying the implants, having received the design files. After approval of the virtual model, the bar was milled from a titanium block, by a five-axis milling machine Newancorvis (Bologna, Italy).

**Fig. 6:**
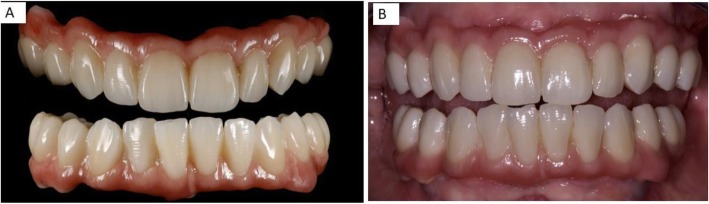
**a** Prothesis completed. **b** Prothesis completed and inserted in the mouth

After being tested in the oral cavity, the bars were delivered to the laboratory to create the zirconia frame using stored data of the temporary and functionalized prosthesis. A trial prosthesis was made on the wax-up, taking into account the aesthetic needs of the patient and, then, a new long-term temporary prosthesis was built, using a systematic CAD/CAM; the advantage of this technique is represented by the possibility of storing useful information that can be used in the next working phases (Figs. 5 and 6). The frame consists of a functional and occlusal part, which is totally anatomical, while the buccal and gingival part is provided with fine ceramic coating (Fig. 6). This protocol does not require any testing of the zirconia frames within the oral cavity, since the correspondence between the model and the mouth was evaluated when testing titanium bars. An Anatomical framework and digital veneering by lithium disilicate and fusion porcelain was produced to reduce the chipping and delamination of the materials. After the final step of ceramization, the prostheses were cemented on the bars with a temporary cement Temp bond (Kerr Italy). The titanium bar was not sandblasted. The large contact surface between bar and prothesis ensures maximum adhesion. The recall visits were done after 6 weeks, 1 year, 2 years, 3 years, 4 years, and 5 years from delivery of final restorations. During the recall visits the stability of the protheses and the integrity of the ceramic of crowns were clinically evaluated. CBCTs were taken at annual recalls and were used to evaluate adaption of bar to the Flatone® abutment.

## Results

In 5 years, no fractures of the ceramic or titanium bars were observed. No other mechanical complications were registered.

At 5 years, no chipping or delamination of ceramic veneering on zirconia-supports were observed. During the observation period, all the crowns were intact, resulting in a survival rate of 100%.

A passive fit of the substructure on the implants was observed in all 18 bars. The passive fit of the titanium bar with multiple individual abutments was confirmed in the oral cavity by periapical radiographs and means of a one-screw test. No gaps were observed at any time between bar and abutment by CBCT. Fifteen finished protesis (93.75%) showed satisfactory occlusion and only one case (6.25%) required significant occlusal adjustment. During the first year recall all bars were stable (100%) no mobility of protheses was recorded. After 5 years all bars were stable (100%) and no mobility of protheses was recorded. During the first-year recall there was no need for re-tightening of occlusal screws. After 5 years only three screws needed re-tightening (3.33%) and occlusal screws were observed in one patient (11.11%). Before loading two implants (2.22%) were removed due to excessive crestal bone resorption and were replaced after 2 months. During the observation period, all implants were asymptomatic without gingival inflammation, resulting in a survival rate of 100%.

## Discussion

The success of an implant surgery depends on the bone and soft tissue healing after the process of implant site preparation [2, 21]. During healing and function of a prothesis the bacterial adhesion is very important for a high success of an implant [22]. Also, the resistance to corrosion of metals for intra oral and dental rehabilitation is important to avoid ions being released as a result of such corrosion [23]. Zirconia is a material used for prosthetic rehabilitation of anterior teeth due to its high biocompatibility, its aesthetic (white color) and mechanical properties [24, 25]. In this case prospective clinical study no chipping nor delamination of ceramic veneering were observed in 5 years. This good result achieved thanks to the marginal fit, which is critical for the success and longevity of a dental restoration [26]. The protocol adopted in this clinical trial leads to a substantial and advantageous reduction in costs and treatment times; it is supported by a high, long-term prognosis that, according to the studies carried out, ranges from 92 to 100% [19–21]. In fact conventional casting techniques are technically difficult for producing a substructure for implant-supported rehabilitation, and the passive fit is difficult in cases of more elements and volume of the substructure [27]. This protocol was recently adopted when restoring implants having unfavorable angulation [28]. The conventional fabrication process of a prosthesis requires multiple materials as well as clinical and laboratory skills. A passive fit of the substructure is necessary for the crestal bone physiology of implants, eliminates overloading of the screw-implant abutments and long term reliability of implant-retained rehabilitation [29, 30]. The Toronto bridges have been used with success in clinical practice for a long time and the only real innovation is the CAD/CAM technology [31] and the data acquisition that can be with or without intraoral scanners. Today the digital technologies and the CAD/CAM technique have an impact on the fabrication of titanium bars for Toronto bridges. CAD/CAM is an important tool for simplifying the prosthetic workflow technologically due to its high-level precision. In dentistry practice several variants have been developed and with them different surgical protocols which, however, are all based on the idea developed by the Swedish school of implantology. One of the main characteristics of this type of prosthesis used in the present prospective clinical study is the flange which reproduces a portion of the alveolar bone and the pink tissue that have been lost by the patient; they become real orthopaedic prostheses, because they do not just replace the teeth, but also the lost bone. The classic Toronto proposed by Brånemark consists of a titanium bar with teeth and resin flange; it is a screwed solution [32, 33]. Although this prosthesis has a good prognosis, it is, unfortunately, not equally valid over time from an aesthetic point of view. Furthermore, the characteristics of acrylic resin do not allow an adequate resistance response when the prosthesis is subjected to a load in the presence of an antagonist other than a full denture. The technique designed by Brånemark consists of a fixed prosthesis on the lower arch, with implants in the intraforaminal area and a removable prosthesis in the upper arch [34]. After this type of prosthetic rehabilitation it is important to maintain hygieniccare of the prothesis. To ensure a professional and domestic hygienic procedure, it is important to avoid flanges on the intaglio and that the intaglio surface of the bridge is convex and hygienic, to plan the proper number of implants to balance support of the bridge with the patient’s ability to keep the bridge clean. Also the materials used to make the prothesis are important. For this reason, in this study Zirconia was used, because it is a material with high biocompatibility [35] and reduces bacterial adhesion [36]. Over the years the use of implants has become more and more common, even for the upper arch and this has resulted in an increase in resin fractures, which occur in 15% of cases; sometimes they are also accompanied by the fracture of the metal structure. Therefore, protocols aimed at finding a solution are constantly changing, in order to improve aesthetic and functional aspects, which are considered essential. Besides choosing the materials, the techniques used for constructing the prosthesis are important. In recent years, computer-aided design (CAD) and computer-aided manufacturing (CAM) have been used in implant rehabilitation. These digital techniques eliminate the need for making an impression using elastomeric impression material. These techniques capture images of the implant, adjacent, and opposing teeth with elimination of the dimensional stability of impression materials and the pouring of stone casts [37]. They use milling restoration from a sintered ceramic block with a very homogeneous structure which improves the quality of the material compared with conventional ceramic processing techniques [38–40].

According to the literature, zirconia crowns cemented on titanium abutments have an excellent prognosis [41]. This technique was used currently in the anterior region of the maxilla, especially when a patient has a thin gingival biotype and it is an aesthetic alternative to other dental materials.

The stiffness typical of this material does not seem to be a problem, because the superstructure is cemented on a titanium bar, which is the gold standard material for this type of prosthesis, as it is soft and has excellent shock absorption. Moreover, the cementation with a material based on zinc oxide ensures good retention but can also be easily removed [42–47]. For the superstructure material zirconia was chosen because it is a material that can replicate, with a fully digital workflow, the data acquired from the diagnostic prosthesis, which makes it possible to use already tested parameters.

Finally, there is a further advantage, that is a reduction of the time required by the laboratory for a similar work, which translates into a reduction of costs for the laboratory, for the clinician and the patient.

## Conclusion

After 5 years clinical observation, this clinical study demonstrates that the zirconia crowns cemented on titanium bars using the CAD/CAM compound prosthesis can be used in clinical practice with high success. Moreover, the computerized workflow of this manufacturing process ensures reproducible results and excellent adaptation and passive insertion of these bars. A condition that guarantees stability of the crestal bone and screw-implant abutments and avoids biological or mechanical complications. In fact, no evidence of chipping or delamination of the ceramic veneering on the zirconia crown supported were observed and a good satisfactory of occlusion are recorded with 100% of implant survival. The importance of the present paper on 5-year results, although on a relatively limited number of patients, reports a restorative principle where mid-term data are absent from the literature. However, due to the limitations associated with the use of only nine case reports and 90 implants, further research is required to confirm this.

## Data Availability

The datasets used and/or analysed during the current study are available from the corresponding author on reasonable request.

## Abbreviations

3D: Three-dimensional
CAD/CAM: Computer-aided design and computer-aided manufacturing
CBCT: Cone-beam computed tomography
